# The Gut–Bone Marrow Axis: Deciphering the Mechanistic Impact of Microbial Metabolites on Hematopoietic Homeostasis and Disorders

**DOI:** 10.3390/microorganisms14071446

**Published:** 2026-06-30

**Authors:** Jiaqi Sun, Yun Ruan, Liming Mao, Lingli Jiang

**Affiliations:** 1 Basic Medical Research Center, School of Medicine, Nantong University, Nantong 226019, China; 2431310020@stmail.ntu.edu.cn; 2 Department of Immunology, School of Medicine, Nantong University, Nantong 226019, China; 3 Department of Gastroenterology, Affiliated Hospital of Nantong University, Nantong University, Nantong 226001, China; roxy@stmail.ntu.edu.cn

**Keywords:** gut-bone marrow axis, hematopoietic system, hematopoietic stem cell, gut microbiota, metabolites, hematologic diseases, dysbiosis

## Abstract

The gut microbiota is increasingly recognized as a dynamic endocrine-like microbial network that exerts systemic effects far beyond the gastrointestinal tract. Emerging evidence supports the existence of a “gut-bone marrow axis” through which gut-derived signals orchestrate hematopoietic homeostasis. However, shifting from correlative observations to causal mechanisms remains a major challenge in defining precise microbial impacts on hematopoietic outcomes. In this review, we systematically synthesize current knowledge on the molecular mechanisms by which microbial products—specifically short-chain fatty acids (SCFAs) and bile acids—translocate into the systemic circulation to modulate hematopoietic stem cell (HSC) function, lineage commitment, and the bone marrow microenvironment. Furthermore, we discuss how gut dysbiosis acts as a driver of hematopoietic dysfunction, contributing to the pathogenesis of anemia, bone marrow failure, and hematologic malignancies such as leukemia. Beyond mechanistic insights, this review critically evaluates the therapeutic promise of emerging microbiota-targeted interventions, including precision probiotics, prebiotics, and FMT, which hold the potential to modulate hematopoietic function and support recovery. Although preclinical evidence is accumulating, these approaches are underpinned by limited yet mechanistically informative clinical evidence. Thus, these emerging interventions require rigorous mechanistic validation and well-designed clinical trials. Herein, by integrating multi-systemic perspectives, we provide a comprehensive framework for future research and clinical strategies aimed at leveraging the microbiota to treat hematologic disorders.

## 1. Introduction

The gut microbiota is a critical component of human health, functioning as a highly interactive and dynamic partner to the host. Beyond its well-established localized roles in digestion and barrier function, the gut microbiota orchestrates a complex, systemic signaling network that transcends the gastrointestinal tract. It plays a key role in modulating the immune system and nutritional metabolism of the host in response to a variety of external environmental factors [1]. Over the past two decades, this understanding has expanded through the description of distinct interorgan networks, including the gut-brain, gut-liver, and gut-lung axes. Their circulating metabolites travel throughout the body to modulate the function of distal organs. These microbial byproducts influence vascular tone and blood pressure stability, while also participating in neural transmission and reproductive endocrine modulation [2]. These widespread and multifaceted effects underscore the indispensable role of the gut microbiota in numerous physiological processes, rather than being compartmentalized in the gut.

Crucially, accumulating evidence has validated that the gut-bone marrow axis serves as a critical module in microbiota-host interorgan crosstalk, mediating bidirectional signal communication between intestinal microbial communities and the bone marrow hematopoietic niche. Hematopoiesis relies on the self-renewal and differentiation of hematopoietic stem cells (HSCs). This process depends on the bone marrow microenvironment, or niche—a specialized cellular architecture of stromal cells, endothelial cells, and osteoblasts that maintains HSC stemness. Specific bioactive metabolites secreted by intestinal commensal microbes enter the systemic circulation and directly or indirectly remodel the proliferation, differentiation, and effector functions of HSCs [3], further highlighting the vital role of gut microbial communities in preserving the structural integrity and normal function of the hematopoietic system.

Consequently, targeted remodeling of gut microbiota homeostasis has emerged as a cutting-edge research field with remarkable translational potential, which is expected to pioneer novel microbiota-targeted intervention strategies for the prevention and treatment of hematological diseases. Although previous studies have extensively discussed microbiota-immune crosstalk within individual metabolic pathways [4], research investigating how gut microbes signal to the bone marrow—the primary niche for immune cell development via systemic microcirculation remains scarce. Within the theoretical framework of the gut-bone marrow axis, this review distinctly shifts the paradigm by systematically elaborating the molecular mechanisms by which the gut microbiota modulates the functions of HSCs and the bone marrow niche, and further explores the prospective translational value of gut microbiota-targeted interventions in the management of disorders associated with hematopoietic dysfunction.

## 2. Composition and Functional Diversity of the Gut Microbiota

The gut microbiota refers to the vast and varied group of microorganisms that reside within the human gastrointestinal tract. This highly complex and dynamic ecosystem is influenced by numerous environmental and host-related factors and consists of trillions of microbes, including bacteria, viruses, fungi, and other microorganisms [5]. Bacteria are the predominant and most extensively studied components of this microbiota. In a healthy individual, the commonly detected bacterial genera include *Escherichia*, *Bifidobacterium*, *Lactobacillus*, *Bacteroides*, and others [6].

The composition of the gut microbiota exhibits pronounced inter-individual variation and spatiotemporal heterogeneity in terms of species diversity, abundance, and relative proportions. Distinct physiological conditions along the gastrointestinal tract shape niche-specific microbial colonization, while external factors, including lifestyle, diet, artificial sweeteners, food additives, and pharmaceutical interventions, continuously remodel the microbial ecosystem [7]. Recent studies indicate that non-nutritive sweeteners [8] and various medications, including proton pump inhibitors (PPIs), metformin, and non-steroidal anti-inflammatory drugs (NSAIDs) [9,10,11], can alter the composition of the microbiota and metabolites. These findings highlight the dynamic ecological plasticity of the gut microbiota during host-microbe co-evolution. Throughout the prolonged history of host-microbial co-evolution, microorganisms have developed highly sophisticated compensatory mechanisms and behavioral regulatory capabilities [12].

However, when pharmacological interventions or environmental stressors exceed the thresholds of ecological tolerance, the conventionally mutualistic microbiota can undergo a symbiotic-to-pathogenic transition, shifting into pathobionts that disrupt pre-existing metabolic interaction networks [13]. Under physiological conditions, the gut microbiota maintains intestinal barrier integrity and produces bioactive metabolites, such as SCFAs and bile acids, which serve as key signaling molecules linking intestinal homeostasis to systemic physiological processes and distant organs.

## 3. The Systemic Reach of Gut Microbiota: Beyond the Gut

The gut microbiota modulates the physiological functions of multiple human systems via the immune-metabolic axis (Table 1). Its microbial metabolites act as pivotal signaling mediators to construct functional crosstalk between the intestine and distant organs, predominantly relying on the circulatory system for systemic signal delivery. In the digestive system, gut microbiota facilitate food digestion and nutrient absorption, and maintain intestinal mucosal barrier integrity by secreting digestive enzymes, synthesizing essential vitamins [14], producing SCFAs [15], and forming protective biofilms [16]. For the immune system, commensal bacteria suppress pathogen overgrowth, promote immune cell maturation, and sustain intestinal immune homeostasis [4]. These metabolites penetrate the intestinal barrier, enter the systemic circulation through the portal vein and liver, and finally reach systemic immune cells and organs. They further mediate the systemic transmission of immune signals through the gut-circulation-systemic immune signaling axis, thereby shaping overall immune homeostasis [17]. While gut microbial metabolites also participate in the remote regulation of other distant organs, such as modulating neurodevelopment or reproductive metabolism [18,19,20], the circulatory system consistently serves as the indispensable central bridge enabling these systemic effects. Specifically, circulating SCFAs like propionate inhibit hepatic cholesterol synthesis [21], whereas indole-3-propionic acid (IPA) facilitates macrophage-mediated reverse cholesterol transport (RCT), modulates myeloid hematopoiesis, and attenuates vascular inflammatory infiltration [22]. Dysregulated production of these core metabolites, particularly the decline in circulating IPA, accelerates the progression of multiple circulatory disorders represented by atherosclerosis [23]. Collectively, this evidence underscores that the gut microbiota mediates extensive intestine-distant organ crosstalk largely through the circulatory system, thereby providing a theoretical foundation for further elucidating its regulatory mechanisms in the hematopoietic system.

## 4. Structural and Functional Basis of Hematopoiesis

### 4.1. Basic Components of the Hematopoietic System

The hematopoietic system is one of the most vital and complex physiological systems in the human body. The hematopoietic system consists of hematopoietic organs, hematopoietic cells, and the hematopoietic microenvironment. During embryonic development, hematopoietic activity is primarily carried out by the yolk sac, liver, spleen, and bone marrow. After fetal birth, the bone marrow becomes the primary organ responsible for hematopoiesis in adults [27]. Hematopoietic cells are composed of a series of mature blood cells and immune cells, including erythrocytes responsible for oxygen transport, megakaryocytes that generate platelets involved in coagulation, as well as bone marrow cells and lymphocytes that play important roles in immune defense. Moreover, blood cells have a limited lifespan. Mature erythrocytes typically circulate for approximately 120 days [28], while platelets survive for about 7–10 days [29].

Hematopoiesis—the continuous process by which HSCs differentiate and mature into red blood cells, white blood cells, and platelets—ensures a constant supply of functional blood cells [30]. HSCs possess two defining properties: self-renewal and multi-lineage differentiation potential. The capacity of self-renewal ensures the stability of the stem cell reservoir during hematopoiesis, thereby sustaining lifelong blood cell production [31]. Multi-lineage differentiation allows HSCs to give rise to different types of blood cells, thereby ensuring the maintenance of normal physiological functions throughout the body. Apart from the multi-wave blood cells that exist from the embryonic stage, which are produced by hematopoietic endothelial cells in the soft yolk sac, all other blood cells originate from HSCs [32].

### 4.2. The Main Stages of Hematopoiesis

**Figure 1:**
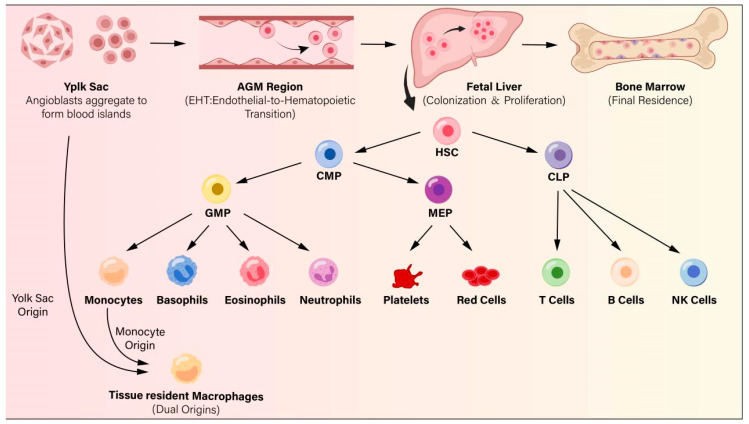
Hematopoietic Differentiation Stages. Early hematopoietic activity initiates in the yolk sac. During embryonic development, hemangioblasts in the yolk sac aggregate to form blood islands, which then migrate to the aorta-gonad-mesonephros (AGM) region, where endothelial-to-hematopoietic transition (EHT) occurs. HSCs subsequently colonize and proliferate in the fetal liver and ultimately establish their permanent niche in the bone marrow. Notably, some subsets of tissue-resident macrophages originate from the primitive yolk sac hematopoietic stage, whereas others are derived from monocytes differentiated from adult bone marrow HSCs. CMP, common myeloid progenitors; CLP, Common Lymphoid Progenitors; MEP, megakaryocyte-erythroid progenitors; GMP, granulocyte-macrophage progenitors. All black solid unidirectional arrows represent progressive differentiation, migration and developmental progression of hematopoietic cells during embryonic and adult hematopoiesis.

The origin of HSCs can be traced back to the embryonic period. In mammalian embryonic development, early hematopoietic activity begins in the yolk sac, an extraembryonic structure [33]. At this stage, the primitive blood cells are not produced by true HSCs. The angioblasts in the yolk sac are common progenitors of both hematopoietic cells and endothelial cells [34]. As embryonic development progresses, angioblasts aggregate to form blood islands (Figure 1). These structures serve dual functions: supporting primitive hematopoiesis, which is independent of defined HSCs, and building the hematopoietic endothelium, which provides the microenvironment necessary for the generation of HSCs. Ultimately, specialized hematopoietic endothelial cells in the aorta-gonad-mesonephros region transform into hematopoietic cells through endothelial-to-hematopoietic transition (EHT), making it the earliest region for the generation of adult HSCs [35]. Around embryonic day 10.5 (E10.5), the emerging HSCs enter the bloodstream and subsequently colonize hematopoietic organs such as the fetal liver, thymus, and spleen. Within the fetal liver, HSCs rapidly proliferate and differentiate into progenitor cells of the erythroid, myeloid, and lymphoid lineages [36]. It is noteworthy that tissue-resident macrophages represent a specialized subset of myeloid-derived cells with heterogeneous hematopoietic origins. Some subsets originate from macrophage precursors generated during the primitive yolk sac hematopoietic stage, which can directly colonize target tissues and self-maintain independently of adult HSCs. Others are derived from monocytes differentiated from adult bone marrow HSCs, which are recruited to tissues before differentiating into macrophages. These cells not only constitute a core component of the local immune microenvironment but also indirectly regulate the differentiation and function of hematopoietically derived immune cells through the secretion of chemokines and inflammatory modulators, thereby contributing to the maintenance of homeostasis within hematopoietic-associated immune microenvironments. Eventually, at E16.5, HSCs migrate to the fetal bone marrow, where they establish residence. This site later becomes the primary site of hematopoiesis in postnatal life. In the adult bone marrow, HSCs maintain lifelong hematopoiesis and are tightly regulated by internal regulatory mechanisms [37].

From the perspective of hematopoietic lineage differentiation, innate immune cells derived from the myeloid lineage, including monocytes, neutrophils, eosinophils, basophils, and dendritic cells, constitute the first line of defense against foreign pathogens [38]. These cells not only provide immediate protection but also help trigger and start adaptive immune responses. In contrast, the lymphoid lineage produces B cells and T cells, which are the core components of adaptive immunity. Lymphocytes mediate pathogen-specific immune responses and play an irreplaceable role in pathogen recognition and clearance [39].

The development and lineage differentiation of the hematopoietic system are not independent processes but have a close bidirectional regulatory relationship with the body’s microenvironment. As the largest microbial community in the body, the intestinal flora can produce metabolites that cross the microenvironmental barrier through the blood circulation and immune signals, act directly or indirectly on HSCs and their various lineages, regulate their differentiation and function, and form a unique gut microbiota-hematopoietic axis.

### 4.3. The Hematopoietic Niche: A Sensitive Sensor for Systemic Signals

The hematopoietic microenvironment, commonly referred to as the hematopoietic niche, serves as a crucial sensory and regulatory hub for HSCs, enabling HSCs to sense and respond to a variety of internal and external signals. In addition to providing essential structural and molecular support for the survival, self-renewal, proliferation, and differentiation of HSCs [40,41], it also functions to integrate multiple physiological signals received in vivo. The HSC niche consists of the extracellular matrix and various stromal cell types such as fibroblasts and macrophages. It not only provides a physical scaffold for HSCs but also secretes a range of cytokines and growth factors to precisely sense and modulate hematopoiesis [42]. Furthermore, the extracellular matrix, composed of structural proteins such as collagen, fibronectin, and laminin, forms a supportive network. Beyond anchoring hematopoietic cells, it also senses microenvironmental alterations and modulates key biological processes of HSCs, including proliferation, differentiation, and migration [43]. HSCs sense endogenous and exogenous stimuli through the microenvironment and adapt hematopoietic activity to alterations in physiological demands of the organism via signaling pathways involved in inflammatory responses and metabolic reprogramming [44].

Additionally, the vascular system in the bone marrow underpins the sensory function of the microenvironment. It not only supplies the necessary nutrients and oxygen required for hematopoiesis but also facilitates the release of mature blood cells into the peripheral circulation. Hematopoietic regulatory factors dynamically interact with HSCs and other components of the hematopoietic microenvironment, enabling the niche to sense physiological fluctuations and thereby maintaining normal hematopoietic function and internal homeostasis [45]. Given that the stability of the bone marrow hematopoietic microenvironment relies on the regulation of peripheral systemic signals, the bone marrow microenvironment serves as a critical target for the regulatory effects of gut microbiota-derived metabolic signals.

## 5. Mechanistic Insights: How Microbes Shape the Hematopoietic Landscape

### 5.1. SCFAs: Direct and Indirect Regulation of HSCs

The gut microbiota-derived short-chain fatty acids (SCFAs), primarily including acetic acid, propionic acid, and butyric acid, serve as key metabolic messengers linking intestinal fermentation to systemic homeostasis [46]. Mounting evidence has uncovered multifaceted links between these microbial metabolites and the hematopoietic system. However, the precise molecular conduits and the cross-species translation of individual SCFAs remain areas of active investigation.

Mechanistically, in vitro and murine studies demonstrate that SCFAs can directly cross biological membranes to act in a cell-intrinsic manner on HSCs and downstream progenitors to modulate their development [47]. Upon entering the bone marrow via the systemic circulation, propionate directly induces specific epigenetic modifications within progenitor cells, namely histone H3 acetylation (H3K27) and propionylation (H3K23). Critically, it selectively reduces the frequency of bone marrow megakaryocyte-erythroid progenitors (MEPs) without affecting the proportions of common myeloid progenitors (CMPs) or granulocyte-macrophage progenitors (GMPs), indicating lineage-specific regulatory activity.

Crucially, this microbiota-driven epigenetic remodeling functions as an upstream regulatory mechanism capable of modulating the expression of various microRNAs (miRNAs) involved in hematopoiesis. SCFAs alter miRNA expression by regulating histone modifications, thereby steering cell lineage differentiation [48]. Notably, miR-126 acts as a key regulator determining the fate of MEPs, where its expression induces the differentiation of these progenitors toward the megakaryocytic rather than the erythroid lineage [49]. Furthermore, miR-223-3p, the most abundant miRNA in megakaryocytes and platelets, serves as a core orchestrator of megakaryopoiesis; its proper expression is indispensable for maintaining correct proplatelet formation and subsequent platelet production [50]. Nevertheless, all these studies share a common limitation: the detailed cellular mechanisms and signaling pathways elucidated therein are almost exclusively derived from in vitro cell culture and animal models, and their direct relevance to human physiological functions remains unvalidated.

Beyond these cell-intrinsic effects, gut microbiota-derived metabolites exert vital indirect regulatory controls over hematopoiesis by actively remodeling the non-hematopoietic bone marrow microenvironment. In murine models of allogeneic hematopoietic stem cell transplantation (allo-HSCT), high dietary fiber intake or prebiotic supplementation with galactooligosaccharides (GOS) promotes the proliferation of butyrate-producing bacteria in the gut and increases intestinal and systemic SCFA levels [51]. Rather than solely targeting hematopoietic clusters directly, the effector molecule butyrate acts via the gut-bone marrow axis to remodel the bone marrow microenvironment. By targeting the bone marrow stromal cells, butyrate coordinates downstream signals to maintain local iron homeostasis in the bone marrow, thereby expanding the supporting capacity of the hematopoietic niche, accelerating hematopoietic reconstitution, and facilitating the functional recovery of HSCs [52]. This stromal niche support effectively safeguards the host’s long-term hematopoietic potential after transplantation. Although dietary or prebiotic interventions exert prominent therapeutic effects in well-controlled mouse models, human patients undergoing allo-HSCT routinely receive broad-spectrum antibiotics and other treatments that drastically remodel the indigenous human microbiota. Under such clinical circumstances, strategies relying on dietary fiber to stimulate endogenous butyrate production may yield minimal therapeutic benefits.

SCFAs also operate through an alternative indirect pathway by licensing and polarizing immune cells, which subsequently secrete systemic or local cytokines that shape the hematopoietic landscape under inflammatory or pathological states. For instance, in experiments where propionate was used to intervene in Collagen-Induced Arthritis (CIA) model mice, it was found that propionate can markedly reduce the levels of pro-inflammatory cytokines such as TNF-α and IL-6 in the serum, thereby shielding the bone marrow from inflammation-induced hematopoietic dysfunction [47]. Similarly, butyrate acts as a potent immune licensor to alleviate inflammation-related damage and improve the survival rate of mice with graft-versus-host disease (GVHD) after allo-HSCT [51,53]. At the immune level, butyrate regulates the NF-κB pathway to suppress excessive T-cell activation, promote regulatory Treg differentiation, and drive M2 macrophage polarization [54]. These licensed immune cells collectively lower systemic inflammatory responses, protecting bone marrow HSCs from inflammatory exhaustion.

In conclusion, butyric acid, propionate, and other SCFAs work together to maintain the balance between hematopoiesis and immunity and alleviate inflammation-related damage in GVHD. They play a crucial role in the interaction between the gut microbiota and the hematopoietic system. Although the feasibility of these effects in human individuals and the standardized clinical administration strategies remain to be deliberated, SCFAs hold promise as an adjuvant therapy for complications of hematological malignancies. To successfully bridge the translational gap between preclinical animal data and clinical therapeutic applications, future research should prioritize human interventional trials and cross-species pharmacokinetic validation.

### 5.2. Bile Acid Metabolism: Influence on the Bone Marrow Microenvironment

Gut microbiota deeply participates in bile acid metabolism through a cascade of enzyme-driven reactions. Primary bile acids secreted by the liver into the colon are first hydrolyzed into free substrates by bile salt hydrolases (BSH) secreted by microbiota such as *Bacteroides* and *Lactobacillus*. These free substrates are then 7α-dehydroxylated by *Firmicutes* via the bai operon to form secondary bile acids, including deoxycholic acid (DCA) and lithocholic acid (LCA) [55,56]. These microbiota-derived bile acids regulate HSCs and the bone marrow microenvironment through two distinct modes of action, which may involve signaling pathways such as the G protein-coupled bile acid receptor Takeda G-protein-coupled receptor 5 [57].

In vitro and murine models demonstrate that secondary bile acids can target HSPCs directly. During embryonic development, fetal liver HSCs utilize bile acids to mitigate endoplasmic reticulum (ER) stress and prevent protein aggregation, thereby sustaining survival and reconstitution capacity [58]. In the adult bone marrow, the secondary bile acid DCA circulates through the bloodstream and directly binds to the vitamin D receptor (VDR) expressed on the surface of bone marrow HSPCs. This interaction markedly upregulates the expression of genes involved in myeloid differentiation and cell proliferation in GMPs, promoting their differentiation into mature myeloid cells while simultaneously inhibiting burst-forming unit-erythroid (BFU-E), ultimately achieving the selective enhancement of bone marrow myeloid hematopoiesis [59]. These direct mechanisms face substantial translational limitations. Marked interspecies disparities exist between humans and mice regarding the developmental timeline of fetal liver hematopoiesis. Additionally, in vitro culture systems fail to fully recapitulate the human bone marrow niche. Moreover, long-term exposure to DCA may elicit intestinal or hepatic toxicities. Collectively, direct in vivo evidence to substantiate the reliability of this proposed mechanism during human embryonic development remains absent.

Concurrently, secondary bile acids exert indirect control over the bone marrow via immune cell modulation. Secondary bile acids (such as tauroursodeoxycholic acid (TUDCA)) produced by gut microbiota metabolism regulate the antigen-presenting function of non-hematopoietic cells and immune cells in the intestine and inhibit apoptosis induced by pro-inflammatory cytokines. Because immune cells are tightly coupled with hematopoiesis, these systemic shifts in immune regulation extend their influence to the hematopoietic microenvironment, thereby indirectly maintaining the stability of the hematopoietic microenvironment and the balance of hematopoietic-related processes [60].

In conclusion, while the gut microbiota-secondary bile acid axis represents a potential metabolic conduit regulating myelopoiesis, current knowledge is tightly restricted to preclinical rodent systems and cell cultures. Prior to clinical translation, additional early-phase human clinical trials are warranted to evaluate the robustness of the underlying regulatory mechanisms.

### 5.3. Other Metabolites: Their Role in Immune Cell Licensing

Due to the diversity of the human gut microbiota, the metabolic products produced are also varied. In addition to SCFAs and bile acids, other metabolic products also influence hematopoiesis (Table 2).

One such compound is IPA, a tryptophan-derived metabolite produced by the gut microbiota. IPA exerts a protective effect on the homeostasis of the hematopoietic system after radiation-induced injury through multiple mechanisms [68]. On the one hand, IPA can maintain the structural stability of the gut microbiota and improve the intestinal barrier function, thereby reducing systemic inflammatory responses and thus creating a favorable internal environment for the normal operation of the hematopoietic system. On the other hand, IPA can specifically bind to PXR binding sites and activate its expression. It reverses the radiation-induced downregulation of acyl-CoA-binding protein (ACBP), thereby promoting the proliferative activity of HSPCs in the bone marrow and alleviating radiation-induced myelosuppression. These findings provide a valuable reference for the treatment of hematopoietic dysfunction caused by radiation exposure.

Citrulline, another metabolic product of the gut microbiota, is produced through the fermentation of bacterial genera such as *Mollicutes_RF39*. As a non-essential amino acid, citrulline mainly participates in the metabolic pathways of arginine and proline. It can influence benzene-induced early hematopoietic toxicity by regulating downstream metabolites such as creatine, 4-hydroxyproline, and glutamate [64]. Purine derivatives represent another class of microbiota-derived metabolites with physiological relevance. Inosine, a key intermediate in purine metabolism, promotes uric acid production when its concentration increases. However, certain *Lactobacilli*, such as *DM9218*, can degrade inosine by expressing inosine hydrolase, thereby reducing hepatic precursors of uric acid synthesis [71]. At the same time, these bacteria also regulate the gut microbiota structure, improve gut barrier function, and decrease levels of lipopolysaccharide (LPS) and inflammatory factors, ultimately enhancing the differentiation ability of hematopoietic stem cells [69].

Although these metabolites have garnered attention for their hematopoietic regulation, most studies are limited to murine models. Consequently, whether animal dosages are applicable to humans remains a key challenge. Moreover, due to the complexity of the human internal environment, positive regulatory mechanisms observed in mice may cause opposite outcomes in humans. Therefore, further clinical trials are essential to validate these findings and prevent potential risks.

### 5.4. Pattern Recognition Receptors (PRRs): TLR/NOD Signaling in Hematopoietic Cells

The gut microbiota can maintain the stability and normal function of HSCs in the bone marrow through an immunoregulatory effect. Systemic translocation of low-level pathogen-associated molecular patterns (PAMPs) from the gut microbiota acts as a tonic regulator of basal hematopoiesis [72]. Under physiological conditions, the microbiota in the gut and the metabolites it produces can regulate the HSC microenvironment by activating Nucleotide-binding oligomerization domain-like receptors (NLRs) [73]. For example, certain substances produced by symbiotic bacteria can activate NLRP3, prompting bone marrow stromal cells to secrete cytokines such as stem cell factor (SCF) and granulocyte-macrophage colony-stimulating factor (GM-CSF). These cytokines help maintain HSC self-renewal and differentiation into myeloid cells, ensuring normal hematopoietic function [74]. In pathological conditions, NLR signaling activated by gut microbiota dysbiosis can disrupt the hematopoietic process. For example, during infection or inflammation, an imbalance in the gut microbiota activates the NOD-like receptor family, pyrin domain-containing 3 (NLRP3) inflammasome, resulting in the release of large amounts of IL-1β and IL-18. These inflammatory cytokines impair the self-renewal capacity of HSCs and promote their excessive differentiation into myeloid cells, disrupting hematopoietic homeostasis.

The gut microbiota indirectly affects the hematopoietic system and the adaptive immune system by influencing the cell differentiation of the hematopoietic system and the expression of immune-related genes in the gut. In vitro evidence has demonstrated that the gut microbiota influences the differentiation of hematopoietic cells. For example, specific microbiota associated with pancreatic cancer can activate TLR2 and TLR5, promoting macrophage polarization toward the immune suppressive M2 type. This reduces the expression of immune-related genes and signaling molecules, inhibiting the activation of CD4^+^ and CD8^+^ T cells, which are key players in both innate and adaptive immune responses, and ultimately leading to immune suppression in the tumor microenvironment [75].

The gut microbiota can also indirectly influence the hematopoietic system through interactions with the immune system. Imbalances in the gut microbiota can trigger an inflammatory response, wherein released inflammatory cytokines enter the bloodstream, affecting the bone marrow microenvironment and disrupting the normal function of HSCs. In inflammatory bowel disease (IBD), dysbiosis of the gut microbiota can lead to neutrophil dysfunction. Many IBD susceptibility genes are involved in the regulation of neutrophil function, affecting their ability to defend against microbes [76]. The abnormal interaction between the gut microbiota, immune system, and hematopoietic system may lead to hematopoietic dysfunction, further exacerbating the progression of the disease.

Therefore, the gut microbiota plays an irreplaceable regulatory role in the development and differentiation of HSCs in the bone marrow, leading to the relevant transitions of lymphoid and myeloid cells, thereby indirectly affecting the development of blood cells and the differentiation of immune cells.

### 5.5. Gut Barrier Integrity and Microbial Translocation (Leaky Gut in Hematopoiesis)

Changes in the gut microbiota structure lead to alterations in the microbiota metabolic products, which in turn affect the intestinal barrier function. SCFAs can indirectly influence pathogen infection by maintaining the integrity of the intestinal barrier and activating intestinal immunity [77]. This barrier plays a critical role in managing a dense population of symbiotic microorganisms while simultaneously defending against invasive pathogens. The permeability of the intestinal barrier can affect the normal function of hematopoietic-related immune cells. Abnormalities in these cells can compromise barrier function and contribute to the onset of intestinal diseases [78]. To maintain homeostasis, the intestinal barrier needs to balance nutrient absorption and regulate water and electrolyte exchange while coping with symbiotic microorganisms and resisting pathogen invasion. Achieving these functions, the intestinal barrier has evolved a complex cooperative relationship between epithelial cells and hematopoietic immune cells.

The intestinal barrier maintains its permeability by sustaining the intact expression of tight junction proteins such as Occludin and Claudin-1, thereby stabilizing the structure of the gut microbiota. Research has found that after exposure to benzene at concentrations of 10 ppm and 50 ppm in mice, the expression of tight junction proteins in their colon tissue was reduced, compromising the integrity of the intestinal barrier and leading to increased intestinal permeability. When the intestinal barrier is damaged, it causes microbiota dysbiosis, which in turn elevates the levels of butyrate and IPA in plasma. However, the mechanistic credibility of these metabolic shifts warrants critical evaluation. Whether these specific microbial alterations are primary drivers or merely secondary byproducts of systemic benzene toxicity remains to be fully decoupled. Abnormal metabolites such as LPS can leak into the bloodstream, triggering a systemic inflammatory response and immune disorders. Through the mode of indirect regulation via immune cells, these disrupted immune cells and elevated inflammatory cytokines act as central regulators that disturb the hematopoietic microenvironment. Concurrently, via the mode of direct action on HSCs, these circulating abnormal metabolites and inflammatory signals directly regulate the proliferation and differentiation of HSPCs. This dual-mode disruption leads to abnormal proportions of HSPC subsets, including LSK (Lineage^-^Sca-1^+^c-Kit^+^) and Multipotent Progenitors, further inducing damage to the hematopoietic system and resulting in a reduction in peripheral blood cells [79]. Since immune cells and cytokines are central regulators of HSC proliferation and differentiation, these findings reveal the strong connection between the gut system, immune system, and hematopoietic system.

## 6. Dysbiosis and Hematopoietic Dysfunction

### 6.1. Reverse Remodeling of Gut Microbiota Diversity and Systemic Metabolism Under Disease Conditions

Traditional research on the “gut-bone marrow axis” has primarily focused on primary gut dysbiosis as an upstream factor, exploring how microbiota alterations induce or exacerbate hematopoietic abnormalities by shifting the host metabolic profile. However, the host’s primary pathological state, malignant tumor infiltration, and immune stress can also retrogradely reshape the gut microbiota and participate in systemic metabolic regulation. This reverse regulation, driven by the host’s pathological condition, not only disrupts the original homeostasis of the gut microecosystem but also establishes a pathological foundation for the gut microbiota to further impair hematopoietic function.

Disruption of physiological barriers and mild inflammation can precipitate a “microbial-metabolic drift.” Under conditions of systemic disease or aging, systemic redox homeostasis is compromised. When the host undergoes systemic disease or aging, systemic redox balance is disrupted, leading to the continuous depletion of peripheral lysophosphatidylcholines (LPCs). Concurrently, toxic microbial metabolites that promote inflammation or enhance susceptibility to ferroptosis accumulate substantially. This metabolic dysregulation drives a subsequent decline in microbial diversity [80].

Furthermore, some diseases trigger systemic immune stress that retrogradely disrupts amino acid metabolism via host signaling pathways. During malignant progression, including multiple myeloma (MM) or high-intensity immunotherapy, sharp fluctuations in circulating inflammatory cytokines and neurotransmitters disturb tyrosine metabolism [81]. This systemic metabolic dysregulation, alongside intestinal epithelial barrier damage, impairs the colonization of beneficial microbes like *Lactobacillus*, leading to altered microbiota composition and the uncontrolled overgrowth of opportunistic pathogens.

This host-driven reverse remodeling of microbiota and metabolism highlights a retrograde feedback mechanism within the gut-bone marrow axis. Rather than being a simple endpoint of dysbiosis, disease acts as a starting point that disrupts gut microbiota balance. This bidirectional regulation plays an essential role in how the gut microbiota unidirectionally drives hematological disorders.

### 6.2. Impact on Benign Hematology: Anemia

**Figure 2:**
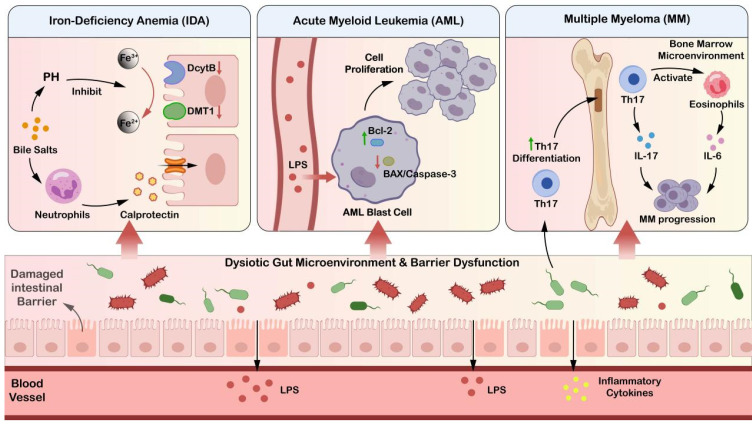
Intestinal flora imbalance leads to the occurrence of hematopoietic system diseases. Gut microbiota dysbiosis and the release of microbiota metabolic products such as lipopolysaccharide (LPS) disrupt the intestinal barrier function, allowing external stimuli to enter the bloodstream, activating immune cells to secrete inflammatory factors. Overproliferation of pathogenic bacteria leads to iron deficiency anemia (IDA) via two pathways: on the one hand, they release harmful metabolites such as secondary bile salts, downregulate the expression of key iron absorption molecules (DcytB, DMT1), and inhibit the conversion of Fe^3+^ to Fe^2+^ by modulating intestinal pH; on the other hand, they trigger intestinal inflammation, promote neutrophils to release calprotectin, and further exacerbate iron metabolism disorders. The diversity of intestinal microbiota is significantly reduced in acute myeloid leukemia (AML) patients. Metabolites produced by pathogenic bacteria impair intestinal barrier function, resulting in massive leakage of LPS from intestinal microbiota into the bloodstream. LPS then promotes AML cell proliferation by upregulating the anti-apoptotic protein Bcl-2 and downregulating the pro-apoptotic protein BAX and cleaved caspase-3, ultimately accelerating disease progression. Intestinal dysbiosis occurs in MM patients. Specific pathogenic bacteria induce the differentiation of Th17 cells and their migration to the bone marrow. Th17 cells secrete IL-17 and activate eosinophils to release IL-6, which ultimately promotes the development of hematological malignancies. Thick red solid arrows indicate downregulation, reduction and inhibitory effects; green arrows represent upregulation, elevation and promotional effects; black solid arrows stand for general biological regulation and induction.

There is a strong long-distance connection between the gut microbiota and the host’s systemic immune response. Dysbiosis disrupts the stability of this relationship, significantly impacting pathogen clearance and inducing pathological immune dysregulation and hematologic malignancies (Figure 2).

Gut microbiota dysbiosis can lead to various hematologic diseases, including anemia, leukemia, and other myelodysplastic syndromes. Anemia, one of the most common diseases of the hematopoietic system, is primarily caused by blood cell loss, impaired utilization of hematopoietic materials, or loss of essential raw materials. Systemic iron deficiency or impaired utilization can damage red blood cell production, thereby triggering anemia [82]. In mammals, iron acquisition is tightly regulated by a comprehensive system controlled by duodenal enterocytes. Therefore, maintaining a balanced gut microbiota is an effective measure to ensure systemic iron balance [83].

Among different types of anemia, iron-deficiency anemia (IDA) is closely related to gut microbiota imbalance. Microbiota dysbiosis promotes the selective overgrowth of harmful pathogenic bacteria such as *Subdoligranulum*, which synthesize iron-chelating siderophores that sequester iron ions in the intestinal lumen and thereby impair their intestinal absorption and utilization. Meanwhile, harmful metabolites such as secondary bile salts are released, disrupting the intestinal barrier, downregulating the expression of key molecules involved in iron absorption, and inhibiting the conversion of Fe^3+^ to absorbable Fe^2+^ by modulating gut pH, thus triggering inflammation. This release of inflammatory factors further interferes with the expression of molecules regulating iron homeostasis, exacerbating iron metabolism dysregulation [84].

In addition to IDA, the gut microbiota plays a similarly pivotal role in regulating the pathways underlying the onset and progression of anemia of inflammation (AI) and anemia of chronic disease (ACD). Microbial imbalance and increased intestinal permeability facilitate the translocation of microbial products and the activation of inflammatory pathways. Pro-inflammatory cytokines, particularly IL-6, stimulate hepatic hepcidin production, leading to ferroportin degradation, reduced intestinal iron absorption, and iron sequestration within macrophages [85]. This dysbiosis-mediated mechanism of hyperhepcidinemia constitutes a critical pathological link in AI and ACD. Given that the human inflammatory milieu involves multi-system regulatory circuits and a far more complex cytokine network, the translational value of findings derived from these highly controlled animal models and in vitro cellular assays remains to be fully elucidated.

Therefore, future research should focus on identifying specific probiotics or metabolites capable of ameliorating gut dysbiosis and suppressing systemic inflammation. Furthermore, targeting the gut microbiota-hepcidin axis warrants evaluation as a promising clinical adjuvant therapy for refractory iron deficiency anemia and anemia of inflammation, provided that the therapeutic efficacy of these preclinical interventions can be validated in prospective human clinical trials.

### 6.3. Role in Malignancies: Leukemia and Other Hematologic Malignancies

Leukemia is a hematologic malignancy that leads to bone marrow dysfunction, often characterized by the abnormal proliferation of hematopoietic stem cells [86]. Gut microbiota and its metabolites, such as SCFAs, can regulate cellular immunity in the bone marrow microenvironment by inhibiting the secretion of inflammatory cytokines. Nevertheless, this immunological equilibrium is vulnerable to gut microbial dysbiosis, a perturbation that emerges as a potential driver of leukemia progression rather than a mere secondary epiphenomenon.

Acute myeloid leukemia (AML) onset and progression are tightly linked to profound gut microbial dysregulation. Observational studies in AML patients revealed a decreased abundance of bacteria such as *Firmicutes* and *Roseburia*, which leads to a reduction in butyrate concentration in the gut. In murine models of AML, this butyrate deficiency disrupts intestinal epithelial tight junction proteins, resulting in increased intestinal permeability and allowing large amounts of microbiota-produced LPS to leak into the bloodstream. Furthermore, when the AML mice were administered sodium butyrate or *Firmicutes*, intestinal barrier repair was observed, which reduced LPS leakage and delayed AML progression [87].

Radiation and chemotherapy administered during allo-HSCT in leukemia patients can directly lead to gut microbiota imbalance, characterized by reduced microbial diversity, decreased symbiotic bacteria, and increased colonization by pathogenic bacteria such as *Enterococcus species*. *Enterococcus species* (e.g., *Enterococcus faecium* and *Enterococcus faecalis*) can damage the intestinal barrier, induce inflammation, activate donor alloreactive T cells, and induce or aggravate acute GVHD. Conversely, bacteria like *Barnesiella*, *Lactobacillus johnsonii*, and *Blautia species* exert protective effects by maintaining intestinal barrier integrity, regulating immune tolerance, and producing SCFAs, thereby alleviating GVHD and improving prognosis. However, the optimal timing and dosage of microbiota-targeted interventions for leukemia transplant patients remain poorly defined, warranting clinical trials to validate efficacy and safety.

In addition to leukemia, common hematologic malignancies include Hodgkin lymphoma, malignant plasma cell tumors, follicular lymphoma, diffuse large B-cell lymphoma, and myeloproliferative tumors. The gut microbiota partially regulates the occurrence and progression of hematologic cancers due to its anti-inflammatory properties [88]. Dysbiosis of the gut microbiota can lead to immune dysfunction and increased inflammatory responses, thereby promoting tumor growth. The composition and abundance of the gut microbiota vary across different types of hematologic malignancies. The susceptibility to malignant plasma cell tumors is positively correlated with the relative abundance of *Eubacterium rectale* in the rectum [89], while an increased abundance of *Clostridium leptum* and *Pseudomonas aeruginosa* has been observed in the gut of MM patients. At the same time, the reduction in SCFA-producing bacteria leads to a decrease in the levels of SCFAs, resulting in the massive release of pro-inflammatory factors. Meanwhile, it induces the abnormal differentiation and migration of Th17 cells and the secretion of IL-17, impairing the tumor-suppressive effect. This promotes the development of hematologic malignancies [90]. Although modulation of the gut microbiota can alleviate hematological cancers to a certain extent, how to precisely regulate the abundance of relevant beneficial bacteria to exert favorable effects on the treatment of these cancers remains a challenging issue.

### 6.4. Bone Marrow Failure and the “Inflammaging” of the Hematopoietic Niche

Microbiota imbalance can impair intestinal barrier function, allowing bacteria and their metabolic products to enter the bloodstream, triggering an inflammatory response. Elevated pro-inflammatory mediators further remodel and drive inflammaging of the hematopoietic niche, which disturbs the normal homeostasis of the bone marrow microenvironment, restricts the proliferation and differentiation of HSCs, and ultimately aggravates the progression of bone marrow failure and myelosuppression. The overgrowth of harmful bacteria, such as *Enterococcus*, produces toxins and inflammatory mediators that affect the normal function of bone marrow cells and suppress hematopoiesis [91]. Microbiota imbalance also affects nutrient absorption, leading to deficiencies in the nutrients required for hematopoiesis, which indirectly impacts hematopoietic function.

Bone marrow failure and myelosuppression lead to a weakened immune system, impairing the body’s ability to effectively defend against invading pathogens. At the same time, the disrupted immune and bone marrow microenvironment further disturbs the colonization and reproduction of beneficial intestinal commensals, deteriorating gut microbiota dysbiosis. In clinical practice, patients with hematopoietic failure and bone marrow suppression frequently experience severe gut microbiota dysbiosis, which increases the risk of infection, complicates treatment, and worsens long-term prognosis. Among patients undergoing HSCT, pre-transplant conditioning-induced myelosuppression and immunosuppression severely disrupt gut microbial equilibrium, thereby aggravating hematopoietic niche inflammaging. This dysbiosis predisposes patients to infections and other complications, thereby negatively affecting the success rate of the transplant and patient survival [91]. Therefore, routine monitoring of gut microbiota profiles and targeted interventions to restore microbial homeostasis, including rational antibiotic administration and probiotic supplementation, are critical for alleviating niche inflammaging, improving hematopoietic reconstitution, and optimizing the clinical outcome of patients with bone marrow failure. The ultimate clinical translatability of these strategies hinges fundamentally on overcoming major clinical bottlenecks, such as establishing standardized protocols for optimal probiotic strains.

## 7. Clinical Implications and Therapeutic Frontiers

### 7.1. Precision Probiotics and Prebiotics in Hematology

Probiotics are defined as live beneficial microorganisms that can be selectively introduced into the gastrointestinal tract through oral administration to colonize the intestinal mucosa of the host and subsequently improve host health. Commonly used strains include *Lactobacilli*, *Bifidobacteria*, and others. The supplementation of multiple probiotics can help improve vascular dysfunction in humans, potentially reducing the risk of hematological diseases (Table 3).

Recent studies have highlighted the immunomodulatory effects of *Bifidobacterium* species, positioning these strains as key regulators of intestinal and systemic immune homeostasis. Notably, these strains are capable of metabolizing human milk oligosaccharides (HMOs), thereby orchestrating a dual anti-inflammatory cascade: they suppress the biosynthesis and secretion of pro-inflammatory cytokines while augmenting pivotal anti-inflammatory mediators including interleukin-27 (IL-27) and interleukin-10 (IL-10). This balanced regulatory framework effectively dampens systemic and intestinal inflammation [17], offering profound translational prospects for the prophylaxis and clinical remission of inflammation-driven hematological conditions, particularly autoimmune hematological disorders. Moreover, it was found that supplementing with the probiotic *Clostridium butyricum* in a mouse model could inhibit the growth of *Citrobacter freundii*, remodel the gut microbiota, and reduce circulating nitrogen levels, ultimately reversing the resistance of MM cells to BTZ. This probiotic-mediated regulation of the gut-bone marrow axis and its impact on MM cell resistance provides a new perspective for the treatment of hematologic tumors [94].

Beyond immunomodulation and chemoresistance reversal, select probiotic strains exert protective effects on the hematological system by preserving intestinal barrier integrity. The probiotic strain *Akkermansia muciniphila* can improve intestinal barrier function through the modulation of tight junction proteins such as occludin and claudins. This effect helps reduce the influx of inflammatory molecules such as LPS into the bloodstream, thereby lowering its adverse effects on the blood system [99]. Additionally, a clinical trial on healthy adults revealed that a 4-week supplementation with *Bacillus subtilis DE111* optimized lipid parameters [100], suggesting that probiotics may beneficially influence lipid metabolism-related pathways to indirectly lower the risk of dyslipidemia-associated circulatory and hematological disorders.

Prebiotics are a class of dietary carbohydrates that cannot be digested and absorbed by the human body, yet can specifically stimulate the growth of beneficial intestinal bacteria, boost the metabolic activity of probiotics, and thereby promote host health. These microbes ferment prebiotics to produce SCFAs, which fulfill energy requirements for colonocytes, optimize the luminal microenvironment, and lower intestinal pH and redox potential to outcompete and restrict pathogen proliferation [101]. A clinical trial demonstrated that the intake of prebiotic resistant starch (RS) preserved microbial diversity and sustained fecal butyrate levels post-transplantation. Concurrently, the administration of a mixed prebiotic formulation (GFO) containing glutamine, polydextrose, and lactulose protected mucosal integrity and alleviated intestinal inflammation, collectively reducing conditioning-induced mucosal damage and lowering the incidence and severity of acute graft-versus-host disease (aGVHD) [96]. Moreover, in Sprague Dawley (SD) rat models, dietary interventions with 5–10% galacto-oligosaccharides (GOS) and 10% fructo-oligosaccharides (FOS) modulated microbial structures to upregulate divalent metal transporter 1 (DMT1) and ferroportin in the colon, facilitating iron absorption and effectively improving symptoms of IDA [97].

However, despite these promising findings from preclinical investigations, the clinical translation of probiotics and prebiotics in hematological disorders remains substantially constrained, with several critical bottlenecks precluding their routine clinical adoption. First, available clinical evidence is extremely scarce and largely derived from small-scale trials or animal models, failing to guarantee reproducible therapeutic effects in human cohorts. Second, stringent safety concerns represent a paramount hurdle unique to hematological practice. Patients undergoing chemotherapy or HSCT frequently develop severe neutropenia and profound immunosuppression; administration of live bacterial strains inherently carries an inherent risk of opportunistic bacteremia and systemic invasive infection. As candidate therapeutic agents rather than over-the-counter dietary supplements, probiotics and prebiotics lack standardized clinical regulatory frameworks, posing formidable obstacles to uniform quality control throughout production and clinical administration.

### 7.2. Postbiotic Interventions for Hematological Disorders

Postbiotics are complex substances consisting of inactivated microbial cells, microbial components, and microbial metabolites, which can exert beneficial functions such as immune regulation and intestinal barrier repair without the administration of live bacteria. As a typical postbiotic, lactic acid produced by intestinal microbiota modulates hematopoiesis through the canonical lactate-GPR81-SCF signal transduction pathway. Lactic acid produced by intestinal lactic acid bacteria enters the circulation and travels to the bone marrow, where it binds to the specific receptor GPR81 on leptin receptor-positive mesenchymal stromal cells (LepR^+^ MSCs) surrounding bone marrow sinusoids. This interaction specifically upregulates the transcription and secretion of stem cell factor (SCF), thereby facilitating the self-renewal and proliferation of HSCs. This signaling axis also facilitates hematopoietic reconstitution following radiation- or chemotherapy-induced myelosuppression, and such regulatory effects are strictly dependent on intact GPR81 signaling [102].

As another type of postbiotic, the cellular components and lysates of *Akkermansia muciniphila* regulate hematopoietic function via the MYD88/TRIF-mediated innate immune signaling pathway after entering the body. In the early stage, the upregulation of granulocyte colony-stimulating factor (G-CSF) and downregulation of C-X-C motif chemokine 12 (CXCL12) drive the migration of hematopoietic stem and progenitor cells (HSPCs) from the bone marrow to the spleen, initiating the primary hematopoietic response. In the late stage, interleukin-1α (IL-1α) secreted by splenic myeloid cells stimulates the proliferation and differentiation of splenic HSPCs, triggering sustained extramedullary hematopoiesis and ultimately remodeling disordered hematopoietic homeostasis [103]. Collectively, distinct postbiotic metabolites and bacterial structural components exert divergent yet complementary regulatory effects on systemic hematopoiesis via tissue-specific stromal or innate immune signaling cascades. Targeting these microbe-derived postbiotic axes may represent a promising therapeutic strategy to alleviate myelosuppression and restore balanced hematopoietic homeostasis in patients receiving cytotoxic radiotherapy or chemotherapy.

### 7.3. Dietary Interventions and the Ketogenic Diet

Beyond targeted microbial preparations including probiotics, prebiotics, and postbiotics, comprehensive reshaping of dietary patterns can indirectly sustain hematopoietic homeostasis by remodeling the composition and metabolic profiles of the intestinal microbiota. As a representative high-fat, low-carb dietary regimen, the ketogenic diet exerts prominent effects on remodeling intestinal microecology. This dietary intervention elevates circulating levels of β-hydroxybutyrate, a class of ketone bodies capable of suppressing the proliferation of intestinal *Bifidobacterium*. Operating through the mode of indirect regulation via immune cells, such alterations further reduce the abundance of pro-inflammatory Th17 cells in the small intestine and visceral adipose tissue, thereby efficiently alleviating systemic chronic inflammation [104]. Since the onset of most hematopoietic disorders is accompanied by inflammatory responses, the reduction in pro-inflammatory cell populations effectively abrogates chronic inflammation-induced damage to the hematopoietic niche, creating permissive conditions for the restoration of disrupted hematopoietic homeostasis.

To date, evidence regarding the effects of the ketogenic diet on hematopoiesis through gut microbiota modulation remains limited. Existing research predominantly centers on its regulatory effects on intestinal microecology, systemic inflammation amelioration, and metabolic intervention [105]. Whether ketogenic diet-induced microbial remodeling and metabolite alterations influence HSC function and bone marrow niche homeostasis via the gut-bone marrow axis remains largely unexplored. Future research should target the gut-bone marrow axis to identify pivotal targets underlying the regulatory cascade linking ketogenic diet, gut microbiota, metabolites, and hematopoiesis, thereby facilitating the clinical translation of ketogenic diet-based therapies. Nevertheless, the clinical implementation of combinatorial regimens integrating these dietary interventions with three other therapeutic modalities—probiotics, prebiotics, and postbiotics—in hematological practice is confronted with multiple prominent challenges. Among these, the substantial inter-individual variability and the absence of robust regulatory frameworks constitute the primary challenges.

### 7.4. Fecal Microbiota Transplantation (FMT) in HSCT

FMT is an intervention technique for treating diseases associated with intestinal dysbiosis, in which the intact intestinal microbial communities derived from the stool of healthy donors are subjected to a series of treatments before being transplanted into the intestinal tract of diseased recipients to reconstruct the normal intestinal microecosystem of recipients. FMT plays a pivotal and multifaceted role in HSCT, exhibiting promising application prospects in restoring aged hematopoietic function, preventing aGVHD, treating HSCT-related hematopoietic dysfunction complicated with GVHD, and facilitating post-chemotherapy recovery. FMT has shown promising potential in rejuvenating aging hematopoietic cells [3].

In patients undergoing allo-HSCT, FMT holds great clinical value in the prevention and treatment of aGVHD. Studies have identified that FMT derived from donors enriched with *Bifidobacterium adolescentis* markedly improves microbiota engraftment efficiency in recipients and effectively reduces the incidence of severe aGVHD. In contrast, FMT from donors dominated by specific *Proteobacteria* species significantly elevates the risk of aGVHD development [106]. For established GVHD, donor-derived FMT also exhibits unique therapeutic potential. Among 15 patients receiving donor-derived FMT, 10 achieved complete clinical remission within one month. These patients presented a marked increase in intestinal microbial alpha diversity, stable engraftment of donor-derived microbiota, and a remarkable elevation in the abundance of intestinal butyrate-producing bacteria [107]. These findings provide evidence for standardized donor selection of FMT after HSCT, which serves as an effective clinical strategy to reduce the risk of aGVHD (Table 4).

In addition, FMT can also facilitate the recovery of patients after chemotherapy and autologous hematopoietic stem cell transplantation. Intestinal colonization of antibiotic-resistant bacteria (ARB) increases the risk of infection and adversely affects clinical prognosis. FMT is capable of effectively regulating intestinal microbiota composition, reducing ARB colonization, lowering the incidence of post-chemotherapy infections, and thereby accelerating patient recovery [108]. However, due to variations in disease severity among patients, the dosage of FMT is difficult to standardize. Furthermore, whether long-term FMT affects immune reconstitution in patients remains unclear. These issues warrant further investigation to optimize therapeutic strategies.

**Table 4:** Clinical Translation Progress and Evidence Evaluation of Core Microbiota Formulations and Dietary Interventions.

Study Type	Study Objects	Interventions	Target Diseases	Level of Evidence	Clinical Translation Progress	Refs.
In Vitro	Infant cohort and in vitro cultured CD4^+^ T cells	Supplementation with *Bifidobacterium infantis EVC001*	Early-life intestinal and systemic inflammation, immune dysregulation	Moderate	Clarified the immunomodulatory pathway that upregulates galectin-1 and suppresses Th2/Th17 cytokines; provides strong early-life immune imprinting evidence for preventing inflammation-driven conditions.	[17]
In Vivo	MM patients and C57BL/6 mouse models	Supplementation with *Clostridium butyricum*	MM	Low-Moderate	Elucidated the “gut-bone marrow axis” mechanism showing that *C. butyricum* reduces circulating ammonium and stabilizes NEK2 protein to reverse chemoresistance.	[94]
	Iron-deficient growing Sprague Dawley (SD) rats (IDA model)	Dietary supplementation with graded doses of GOS or 10% FOS for 21 days	IDA	Low-Moderate	Demonstrated that prebiotics enhance iron status by remodeling the gut microbiota and upregulating colonic iron-transport proteins.	[97]
	Wild-type, *Gpr81*^−/−^ mice, and irradiated-induced myelosuppression models	Oral administration of lactic acid-producing bacteria or lactate treatment	Radiation or chemotherapy induced myelosuppression, hematopoietic injuries	Low	Discovered that microbiota-derived lactate travels via circulation to bone marrow, binding to GPR81 to trigger SCF production, driving hematopoietic and erythroid reconstitution.	[102]
	C57BL/6 mice and *Myd88*^−/−^, *Trif*^−/−^, *Tlr2*^−/−^, *Tlr4*^−/−^ models	Systemic injection of *Akkermansia muciniphila* cellular components or lysates	Hematopoietic homeostasis disruption, myelosuppression post-cytotoxic therapy	Low	*Akkermansia muciniphila* components trigger delayed extramedullary hematopoiesis (EMH) via MYD88/TRIF signaling, driving splenic myeloid cells to secrete IL-a to stimulate HSPCs.	[103]
	Healthy human, mouse models, and Il17a-gfp reporter mice	Ketogenic diet	Hematopoietic microenvironment injury induced by systemic chronic inflammation	Low-Moderate	KD elevates β-hydroxybutyrate to suppress *Bifidobacterium*, indirectly reducing pro-inflammatory Th17 cells to alleviate systemic inflammation.	[104]
	Aged mice (20 months old) and young mice (2 months old)	FMT from young donors into aged recipients	Age-related HSC dysfunction, myeloid lineage skewing	Low	Young FMT restores lymphoid potential, enhances engraftment of aged HSCs by suppressing bone marrow inflammation via tryptophan metabolites and FoxO pathway.	[3]
	Subcutaneous and orthotopic murine colorectal cancer (CRC) models	Hypoxia-targeted engineered *E. coli Nissle 1917*	Tumor immunosuppressive microenvironment, chemoimmunotherapy resistance	Low	This engineered probiotic converts immunosuppressive adenosine to inosine, promoting a shift from M2 to M1 macrophages.	[109]
	Dextran sulfate sodium (DSS)-induced murine ulcerative colitis (UC) models	Oral administration of propionate-producing genetically engineered probiotics	Murine UC, intestinal inflammation and epithelial barrier disruption	Low	Engineered probiotic derived propionate binds to GPR81/HDAC1 to restore anti-inflammatory macrophages and upregulate IL-10, successfully alleviating colitis.	[110]
Clinical Trial	Healthy adults	Daily oral administration of *Bacillus subtilis DE111* for 4 weeks	Lipid metabolism disorders, systemic chronic inflammation	High	Optimization of lipid parameters and modulation of peripheral blood mononuclear cell (PBMC) immune responses; serves as a ready-to-use strategy via metabolic pathways.	[100]
	Recipients undergoing allo-HSCT	Dietary intake of prebiotic resistant starch and a mixed prebiotic formulation GFO	aGVHD, transplant-related mucositis, and diarrhea	High	This approach preserves intestinal mucosal integrity, reduces the incidence and severity of aGVHD, and maintains intestinal microbial diversity post-transplantation.	[96]
	Patients undergoing allo-HCT	Early high-dose FMT after allo-HCT using different healthy donors to assess donor effects	Prevention of severe aGVHD	Moderate	Donor enriched with *Bifidobacterium adolescentis* dramatically improved engraftment and clinical outcomes.	[106]
	Patients with steroid-dependent acute intestinal GvHD after allo-HCT	Infusion of fecal suspension from unrelated healthy donors via nasoduodenal route	Steroid-refractory or steroid-dependent acute intestinal GvHD	Moderate	Recovery of intestinal flora diversity and significant expansion of butyrate-producing bacteria in patients enabled dose reduction of immunosuppressants in partial subjects.	[107]
	MM patients undergoing autologous stem cell transplantation (auto-SCT)	Clinical assessment of spontaneous gut colonization by antibiotic-resistant bacteria (ARB)	Infections post-auto-SCT in MM patients, gut microbiota dysbiosis	Moderate	Pre-transplant ARB colonization significantly doubles postoperative infection rates and delays neutrophil hematopoietic reconstitution in patients, which inversely confirms the importance of microecological clearance.	[108]

### 7.5. Engineered Microbes for Targeted Delivery of Metabolites

Metabolites derived from native intestinal microbiota are generally characterized by poor in vivo stability, easy degradation, weak targeting ability, and short duration of action. Simple dietary intervention or fecal microbiota transplantation fails to achieve precise enrichment and sustained efficacy in the intestinal tract and cannot specifically modulate the hematopoietic system, which greatly restricts the translational application potential of gut microbial metabolites in hematopoietic system diseases. Fortunately, microbial engineering technology has broken through these bottlenecks.

Safe intestinal strains such as *Escherichia coli Nissle 1917* can be artificially modified via genetic editing technologies to optimize in vivo metabolic pathways and enhance the synthesis and secretion of SCFAs and other functional metabolites. Through functional modification and introduction of environment-responsive elements, engineered bacteria are endowed with the capacities of targeted colonization in the intestine or tumor tissues, response to hypoxic microenvironments and inflammatory signals, as well as controllable release of active substances, thereby constructing engineered microbial vectors for targeted metabolite delivery [109]. These microbial vectors can stably colonize the intestinal tract or lesion sites and continuously secrete functional metabolites. They further modulate immune-related pathways such as macrophage polarization and histone deacetylase signaling via the gut-immune axis, remodel the local immune microenvironment, and regulate inflammatory cytokine secretion and inflammatory homeostasis [110]. Compared with the administration of free metabolites, engineered microbes possess prominent superiorities, including persistent colonization, long-term sustained release, high targeting property, and low side effects, which meet the long-term therapeutic demands for chronic immune imbalance and inflammatory hematopoietic injury related to hematopoiesis. Nevertheless, the biosafety and colonization controllability of engineered microbes still require further systematic evaluation. Collectively, they are expected to emerge as a novel microecological strategy for targeted regulation of the immune-hematopoietic axis and intervention against immune imbalance-related hematopoietic disorders.

## 8. Current Challenges and Discussion

Although the gut-bone marrow axis opens critical investigative paths, bridging the gap between correlative clinical associations and precise mechanistic validation remains a major bottleneck. A pivotal question currently unresolved is whether gut dysbiosis serves as a primary, upstream driver of hematopoietic dysfunction or merely manifests as a secondary, systemic consequence of hematological malignancies, chronic inflammation, and chemotherapy. This complexity is further compounded by the vast “dark matter” within the gut microbiota, as the majority of microbial metabolites and their synergistic networks remain uncharacterized, severely limiting the identification of specific bioactive molecules responsible for regulating HSCs.

Furthermore, the heavy reliance on germ-free or antibiotic-depleted mouse models introduces significant translational friction. Murine and human hematopoiesis differ fundamentally regarding HSC surface markers, lineage bias kinetics, and the cellular architecture of the bone marrow stromal niche. Consequently, whether microbial phenotypes and therapeutic efficacies observed in strictly controlled experimental animals can be recapitulated in highly heterogeneous human populations remains controversial.

When exploring the regulatory mechanisms mediated by metabolites such as SCFAs and bile acids, numerous in vitro and in vivo studies frequently employ supraphysiological concentrations to elicit observable hematopoietic alterations. However, in the steady state, these metabolites exist at low and fluctuating concentrations in the peripheral circulation, leaving it unclear whether they can successfully cross the blood-bone marrow barrier to exert identical regulatory effects. These unresolved issues, combined with the current lack of standardized experimental protocols for metabolites, underscore the critical necessity of addressing these fundamental obstacles before microbial therapeutics can be translated into clinical hematological applications.

## 9. Future Directions

Future research regarding the gut-bone marrow axis should consider shifting from superficial taxonomic profiling toward integrated multi-omics network investigations. Synchronized analyses combining metagenomics, metatranscriptomics, and metabolomics with host single-cell RNA sequencing (scRNA-seq) and spatial transcriptomics are required to map the precise molecular pathways through which distinct microbial metabolites translocate across the intestinal barrier to govern signaling cascades, epigenetic modifications, and metabolic rewiring within the bone marrow microenvironment. Concurrently, identifying specific gut microbial signatures and metabolic profiles capable of predicting functional abnormalities in hematopoietic cells and systemic immune homeostatic imbalances represents another critical avenue. Although dysregulated microbial architectures are frequently observed during gut dysbiosis, current studies predominantly confirm their correlation with hematological pathologies rather than validating their efficacy as reliable predictive indicators. Establishing and validating these non-invasive, microbiota-based biomarkers will enable clinicians to forecast disease progression, optimize targeted therapeutic strategies, and anticipate severe complications—including aGVHD and chemoresistance—well before clinical onset.

Furthermore, future clinical paradigms could pivot toward personalized strategies, such as seeking customized combinations of prebiotics, probiotics, and postbiotics, formulating specialized dietary regimens, or identifying specific donor profiles for FMT. These interventions must be tailored to a patient’s unique baseline microbial composition, metabolic rate, and specific hematological status to maximize efficacy and prevent therapeutic failure driven by inter-individual variability. Crucially, large-scale, randomized, double-blind, placebo-controlled trials are urgently needed. Such studies must explicitly evaluate the long-term efficacy and safety of microbiome-targeted therapies in immunocompromised hematological patient populations, thereby providing robust clinical evidence to safely integrate these microbial interventions into standard hematological guidelines.

In summary, by integrating the findings from gut microbiota composition analysis, metabolite profiling, and HSC functional assessments, we can clearly elucidate how the gut microbiota-hematopoiesis axis regulates the hematopoietic system and immune homeostasis through a complex immunomodulatory network. The discovery of these mechanisms will provide a novel theoretical foundation and feasible practical directions for developing targeted, non-surgical therapeutic strategies, ultimately improving the clinical outcomes and quality of life for patients with severe hematopoietic disorders.

## 10. Conclusions

This review, transcending the conventional scope of peripheral immune regulation, synthesizes the existing research advances that identify the gut microbiota as a key regulator of the hematopoietic system and constructs a framework for the gut-hematopoietic axis that links microbial composition, metabolic signaling, the hematopoietic microenvironment, and stage-specific hematopoietic regulation. By integrating current studies on gut microbiota-mediated hematopoietic modulation, it summarizes the pathological mechanisms of gut microbial dysbiosis in the development of hematopoietic dysfunction and the translational potential of microbial interventions for hematologic diseases. The gut microbiota represents a promising actionable target for regulating mammalian hematopoietic function. Future research may focus on longitudinal human studies to validate the causal relationships between the gut microbiota and hematopoiesis and advance microbiota-directed therapies for hematologic diseases by harnessing the synergistic effects of gut microbiota modulation and existing therapeutic regimens for hematologic disorders.

## Figures and Tables

**Table 1:** The roles of the gut microbiota in various systems.

System	The Functions of the Gut Microbiota	Study Type	Experimental Model/Subjects	Ref.
Digestive system	Regulates the intestinal microbiota structure, enhances the intestinal barrier function, and balances the ratio of Th17/Treg cells.	In vivo study	Ovariectomized (OVX) rat model (non-gene knockout)	[16]
	Through metabolic engineering of *Escherichia coli*, optimizes the vitamin B6 biosynthesis pathway, increases vitamin B6 production, and provides a potential microbial source for vitamin B6 supplementation in the human body.	In vitro study	*E. coli* (strains MG1655, BW25113, etc.)	[14]
Immune system	Gut microbiota dysbiosis and impaired short-chain fatty acid (SCFA) production induce the expansion of Tregs and regulate the secretion of cytokines, forming an immunosuppressive microenvironment.	In vivo and in vitro studies	In vivo: Human subjects (Non-NAFLD controls, NAFLD patients, NAFLD-HCC patients); In vitro: Peripheral blood mononuclear cells (PBMCs) from non-NAFLD controls	[24]
	Gut microbiota dysbiosis affects inflammatory cytokines through regulating metabolic products such as carbohydrates, and participates in the gut-lung immune response.	In vivo study	Human subjects (COVID-19 patients, non-COVID-19 controls)	[4]
Nervous system	Regulates the tryptophan metabolic pathway, affects neurotransmitter balance, and participates in the pathogenesis of neuropsychiatric diseases.	In vivo study	Mouse models (including gene knockout mice: *Card9*^−/−^, *Ido1*^−/−^, *Tph1*^−/−^, *Sert*^−/−^, *Ahr*^−/−^) Human subjects (patients with inflammatory bowel disease, autism and depression)	[25]
Reproductive system	Microbial metabolism produces 3-hydroxyanthranilic acid, which upregulates GPX4 expression, inhibits ferroptosis, and restores spermatogenesis in aged mice.	In vivo and in vitro studies	In vivo: C57BL/6J mice (non-gene knockout); In vitro: GC-2 spd cells	[19]
	Gut microbiota dysbiosis reduces bile acid levels, impairs vitamin A absorption, and causes spermatogonial cell differentiation arrest.	In vivo study	Mouse models (non-gene knockout) and sheep models	[26]
	Gut microbiota dysbiosis affects oocyte quality and embryo development.	In vivo study	Mouse models (non-gene knockout)	[20]
Circulatory system	Gut microbiota metabolism produces propionate (PA), which increases the number of intestinal regulatory T cells and IL-10 levels, inhibits intestinal cholesterol transporter NPC1L1, reduces cholesterol absorption, lowers blood lipids, and alleviates atherosclerosis.	In vivo and in vitro studies	In vivo: *Apoe*^−/−^ mice (gene knockout); human subjects (patients with hypercholesterolemia); In vitro: Mouse small intestinal epithelial organoids	[21]

**Table 2:** Classification of metabolite-mediated effects on the human body.

Classification	Metabolites	Microbiota	Functions	Ref.
Short-chain Fatty Acids	Acetic acid	*Bacteroides* *Bifidobacterium* *Roseburia* *Lactobacillus* *Porphyromonadaceae*	Regulate intestinal immunity, enhance the intestinal barrier function, and maintain hematopoietic-immune balance.	[61] [62]
	Propionic acid			
	Butyric acid			
Vitamins	Vitamin B-group vitamins	*Bifidobacterium* *Lactobacillus*	Take part in blood clotting, energy metabolism, and the maintenance of nervous system function.	[63]
Amino Acid Metabolites	Citrulline	*Mollicutes_RF39*	Affect the intestinal barrier function, regulate the immune response, and promote the proliferation and differentiation of hematopoietic progenitor cells.	[64] [65]
	γ-glutamylalanine	Gut microbiota community		
Bile Acid Metabolites	Secondary Bile Acids (such as DCA and LCA)	*Clostridium* *Bacteroides* *Lactobacillus*	Promote hematopoietic function, regulate the intestinal microbiota, and participate in metabolic regulation.	[66] [55] [56] [59]
Neurotransmitters and neuroactive substances	GABA, Serotonin, 5-hydroxytryptophan, histamine	*Roseburia intestinalis*	Influence the nervous system function via the gut-brain axis, regulate emotions, cognition, and also take part in the regulation of intestinal motility and secretion.	[67]
Other metabolites	Polyamines, barteriocin, indices and purine	*Lactic acid bacteria DM9218*	Maintain epithelial renewal, anti-decay, enhance the intestinal barrier, and promot the recovery of HSCs and progenitor cells.	[68] [69] [70]

**Table 3:** The role of gut microbiota in hematologic diseases.

Category	Mechanism	Effects	Examples	Ref.
Probiotics	Promote the proliferation of beneficial bacteria, regulate gut barrier function, and reduce inflammation.	1. Immune system activation and anticancer effects 2. Adjuvant therapy for leukemia 3. Alleviating resistance in MM cells	*Bifidobacteria* *Lactobacillus rhamnosus* *Clostridium butyricum*	[92] [93] [94]
Prebiotics	As a substrate for the gut microbiota, it promotes the proliferation of beneficial bacteria, regulates gut barrier function, and lowers inflammation.	1. Prevention of intestinal complications associated with HSCT 2. Improvement of intestinal iron absorption in patients with anemia	FOS, GOS, Inulin, Resistant Starch, Euglena gracilis paramylon, Glutamine-Fiber-Oligosaccharide(GFO)	[95] [96] [97]
Synbiotics	Synergistically promote the proliferation of beneficial bacteria, enhance immune modulation, and gut barrier function.	1. Alleviating gastrointestinal side effects after chemotherapy for acute lymphoblastic leukemia (ALL)	LactoCare Synbiotic (containing *Lactobacillus acidophilus*, *Bifidobacterium longum*, etc. + FOS),	[98]

## Data Availability

No new data were created or analyzed in this study.
